# Supplementary figures and images for: Impact of a human gut microbe on Vibrio cholerae host colonization through biofilm enhancement
Source: eLife. 2022 Mar 28;11:e73010. doi: 10.7554/eLife.73010 (PMC8993218; doi:10.7554/eLife.73010)

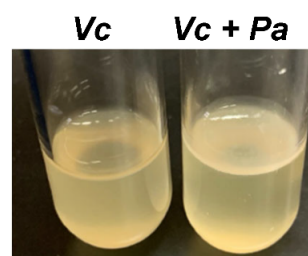

Supplement: Figure 3—source data 2. [file elife-73010-fig3-data2.pdf]
